# Supplementary material for: Patterns of Intron Gain and Loss in Fungi
Source: PLoS Biol. 2004 Nov 30;2(12):e422. doi: 10.1371/journal.pbio.0020422 (PMC532390; doi:10.1371/journal.pbio.0020422)
Supplement: Table S1 — Also available at http://genes.mit.edu/NielsenEtAl/. (4.3 MB ZIP). [file pbio.0020422.st001.zip › NielsenEtAl/html/919.html]

AN3730.1.NCU08909.1.MG08370.1.FG02022.1


```
 CLUSTAL W (1.82) Multiple Sequence Alignments - Introns Inserted


Sequence 1: AN3730.1	542 aa
Sequence 2: FG02022.1	541 aa
Sequence 3: NCU08909.1	542 aa
Sequence 4: MG08370.1	533 aa
Alignment Length: 559 aa
Number Identitical Residues: 182 aa
Alignment Score (without introns) 10226


MG08370.1 	MSFIKLAKALGVLQLVSAAAAATEPVTAK~GQYLFF-KNGTQF~YIRGVAYQQ~GVNVNG
NCU08909.1	MVTSKAAIVA-GLTLAGQASAALQPIVMK0GSKFFY-ENGTQF~YIKGIAYQQ~DAAGAG
FG02022.1 	MKYSAVIVAA-AAAAAS---AKLEPITMK0GSKLFY-TNGTQF~FMKGVAYQQ~DSAAGG
AN3730.1  	MKFSSILAGA-AFFASSVVTADLDPIVIK0GSKFFYKSNDTQF2YIRGVAYQQ1EYS--G
          	*               .  :*  :*:. * *. :*:. *.*** :::*:****      *

MG08370.1 	QGDATSANINDPLADEASCKRDAPLLSKLGTNVIRVYAIDPKKDHTACMKILDNAGIYVI
NCU08909.1	GETTTGT-FKDPLADEAACKRDVPIMAAAGTNAIRTYAIDPTADHSACMKLLDEAGIYVI
FG02022.1 	ESTGKKT-YVDPLADEKACKRDVPLLKELGTNIIRTYAIDPTADHSACMKLLDDAGIYVI
AN3730.1  	PKSDTNN-YKDPLADVEACKRDVPYLQKLNANTIRVYAVDPKADHKECMSLLSDAGIYVI
          	    .     *****  :****.* :   .:* **.**:**. **. **.:*.:******

MG08370.1 	ADLSAPDISVERDSPSWDVSLLARYTGVIDNLSKFENTLGFFAGNEVTNNATNTQASAFV
NCU08909.1	SDLSEPSTSINRDDPRWDITLYKRYTAVIDEMSKYSNVIGFFAGNEVSNNATNTDASAYV
FG02022.1 	SDLSEPSLSINRDDPKWDIELYQRYVDVVDELGQYDNVVGFFAGNEVSNNVSNTQASAFV
AN3730.1  	ADLSSPSESIIRNDPKWDFDLYQRYASVVDELSQYSNVIGFFAGNEVSNDPETTDASAFV
          	:*** *. *: *:.* **. *  **. *:*::.::.*.:********:*:  .*:***:*

MG08370.1 	KAAVRDSKAYIKSKNLGRWLGVGYATNDDSGTRDNLANYFNCGKPEDSVDFWGYNIYEWC
NCU08909.1	KAAVRDSKNYIKNNVK-RWMGVGYAANDDAKIRSEMAHFFNCGNQSEAIDFWGYNIYEWC
FG02022.1 	KAAVRDTKKHLQSKFK-RWLGVGYASNDDVDIREDIADYFNCGEDDSRIDYWGYNIYSWC
AN3730.1  	KAAVRDMKRYIKAKNY-RPMGVGYATNDDSSIRVDMADYFNCGEEEDSIDFWGYNVYSWC
          	****** * ::: :   * :*****:***   * ::*.:****: .. :*:****:*.**

MG08370.1 	GKSTFQSSGYEERTKFFQDYSVPVFFSEYGCQNPGGAAARIFQETGVLYSND-MNKVWSG
NCU08909.1	GHNTIKGSGYQDQIDFFKNYSVPVFFAEYGCNIPDGADGRIFEETTALYSDA-MTDVFSG
FG02022.1 	GKSSMKDSGYANQAEFFKDYSVPVFFAEYGCNEPDGAAGRIFDETTALYEEKVMTDVFSG
AN3730.1  	GDSNYEKSGYKSRTEEFKDYSIPVFFAEYGCN---AVTPRKFTEVEALYGDK-MAEVWSG
          	*... : *** .: . *::**:****:****:   ..  * * *. .** :  * .*:**

MG08370.1 	GIVYEYFEEQNDYG1LVSLS-GNSVTTMKDFGALATQIAQSGYPTGTAMNSYQPSN-TAR
NCU08909.1	GIVYMYFEEDNDYG~LVKVS-GNSAKTMKNYDKLKANVLAAK-PKTVELDSYKPAN-EPA
FG02022.1 	GIVYMYNQEANDYG~LVKISKNGDAVKLKDYSQLQKKANAAN-PQGVDKDDYKPTNDKAA
AN3730.1  	GIVYMYFQEDNNYG1LVSIN-NGNAKTLEDFSYLSKQLASAT-PSGTKKADYNPTNTALE
          	**** * :* *:** **.:. .... .::::. *  :   :  *  .   .*:*:*    

MG08370.1 	PCPAIGDKWKAA-EKLPPSPNSTVCSCMYQSLSCVPSAKTASDAKSIGALFGTVCG-MDP
NCU08909.1	ECPDVTSNWQVTGDALPPTPNESLCECMYNSLTCVPSKDVK--PTAYGELFGVVCG-NDP
FG02022.1 	TCPSQSKSWKAN-SALPPVPDKTLCDCMVKSRSCVPADNLK--AKDFNEIFGYICG-QNK
AN3730.1  	SCPTTGKKWLAAASPLPPSPNSDLCSCMEKSLSCVAKSDIS--GKKLSSTFSTVCGYQGG
          	 **   ..* . .. *** *:. :*.** :* :**.  .     .  .  *. :**  . 

MG08370.1 	KACTGITADAATGTYGLFSMCNATEKLGNVLNAYYN---NQGKSSSACDFKGQASVVASP
NCU08909.1	AACAGIQGNVSTGVYGSYAMCNSLQQLGYVMDQYYK---NQKYASTACDFNGQASANGAA
FG02022.1 	KICTAINANATAGIYGAYSMCDNESKLAYILDAYYK---DQNSNADACDFKGKATTQKAQ
AN3730.1  	KFCEGVSGNATTGKYGAYSVCTPKQQLSFAMNQYYEAQAEQGNGQDACDFDGAATSQSTS
          	  * .: .:.::* ** :::*   .:*.  :: **::.::*     ****.* *:   : 

MG08370.1 	KSDGTCTSVLSAA----------SSAAASGGSGSGSGKNAAGTLRVGAGWGAAFEG----
NCU08909.1	KADPTCSAALKSA--EAANKVAATATAGHGNGGVDGSSGGAAATSSSFAAPVAMKS-FFT
FG02022.1 	N-QDSCKTKLESA--DKTNEEVATATAAAGAASTDGSDSSSKDDDESFGLESATIARAFS
AN3730.1  	EPTAACSSLLSQAGSDGTGSVKASPTAASGGSGGSGDAGADTSEGAAFALSPGSVH----
          	:.  :*.: *. *.:. :..  ::.:*. * .. ... ..      . .   .       

MG08370.1 	--AFGAG-IVVLSAVVGAAAVLM
NCU08909.1	VADMAVGMYVVIAMGVGAGMVML
FG02022.1 	VGNYAVGAYMAVAGVVGAGMVLL
AN3730.1  	VGVFQVGAYVTTAIFAGAAMILL
          	 .   .*  :. :  .**. :::
```
